# Near infra-red absorption spectroscopy for astrophysically significant ions

**DOI:** 10.1007/s10686-025-10009-9

**Published:** 2025-07-17

**Authors:** Kirsten Dowd, Eric Doyle, Padraig Dunne

**Affiliations:** School of Physics, University College Dublin, Dublin, 4 Ireland

**Keywords:** Photoabsorption, Laser-produced plasma, Kilonova, Atomic structure, Laboratory astrophysics

## Abstract

We present a novel laboratory astrophysics experiment to obtain photoabsorption spectra of neutral and near neutral atomic species in the near infrared (NIR) spectral region. A laser produced plasma containing the ions of interest is probed by the collimated output of a supercontinuum fiber laser. The resulting absorption spectrum is recorded on a 0.75-m spectrograph equipped with a complimentary metal oxide semiconductor (CMOS) camera. Spectra of yttrium plasmas were recorded 11 $\upmu$s after its formation in the range from 700 to 1100 nm, and we present the spectrum between 708 to 832 nm to illustrate the capabilities of the technique. In this range we found 26 lines previously identified and 29 lines not previously identified. The importance of new atomic structure data, in particular transition energies and relative oscillator strengths, is highlighted in the context of increasingly sophisticated ground and space-based spectrometers in the era of multi-messenger astronomy. Future developments and improvements are briefly discussed.

## Introduction

Atomic structure determination, based on experimental measurements and complementary theoretical calculations, is a key component in laboratory astrophysics, which is a foundation of modern astronomy [1, 2]. A brief study of the line and level holdings of the Atomic Spectra Database at the National Institute for Standards and Technology (NIST) [3] shows the incomplete nature of the atomic data for most ionised species. In particular, many holdings show few or no spectral lines between 400 and 2000 nm for near-neutral species. This is due to sparsity in the spectra of some ions, but mainly due to a lack of recorded and analysed spectra in many cases.

The visible and near infra-red regions of the electromagnetic spectrum provide broad windows through the Earth’s atmosphere that allow ground-based instruments such as X-Shooter on the Very Large Telescope (VLT) at the European Southern Observatory [4] to record spectra with resolving power $\mathrm {\lambda }$ / $\mathrm {\Delta \lambda }$ of greater than 4,000. Many space-based instruments also have medium-resolving power spectrographic capabilities in this region, most recently NIRSpec on the James Webb Space Telescope [5]. Such current and future developments in the visible and near infra-red raise the need for new methods in laboratory astrophysics to generate and interpret the spectra of near neutral ions. The gravitational wave detection by LIGO and Virgo of GW170817 [6], a binary neutron star (BNS) merger event, led to follow-up studies of the kilonova AT2017gfo using the X-Shooter spectrograph at the VLT, the Hubble Space Telescope (HST), and multiple other observatories. The first signature of an r-process element in the spectrum of AT2017gfo was that of strontium [7]. Simulations predict that neutral and lowly-ionised atomic species will form the bulk of the matter in the kilonova in the days following the merger [8]. While extensive analysis of AT2017gfo has been published, see Gillanders et al. [9] and references therein, the absence of basic atomic data for many candidate species is frequently highlighted [10]. These data include atomic energy levels, oscillator strengths and wavelengths, which are crucial in the creation of radiation transport models, in the interpretation of observed spectra, and connecting back to the underlying merger physics.

Photon-based spectroscopy techniques, such as photoabsorption, photo-electron, and photo-ion spectroscopies, are key techniques in the evaluation of atomic structure [11]. Photoabsorption has advantages compared to emission spectroscopy in probing ground state systems in relatively low temperature regimes (5,000 K - 20,000 K) and in probing metastable states. It is particularly suited to recording accurate relative oscillator strengths, as many transitions arise from ground-state energy levels, or low-lying excited states, with similar populations. At higher photon energies (i.e. above ${\sim }$10 eV), photoabsorption studies facilitate probing inner shell structure and dynamics. Experiments operate in either pulsed or continuous mode, mainly dictated by the source of the absorbing species, but also the nature of the continuum radiation source used. Pulsed experiments allow enhanced selection of ion populations by synchronising the probe pulse to a particular phase in the temporal evolution of the source of the species of interest. A wide range of experimental approaches are based on probing atomic vapours with tuneable synchrotron radiation [12], evolving to more sophisticated experiments merging ion beams and synchrotron radiation in photoionisation studies [13]. These experiments typically require large-scale facilities and complex apparatus to generate sufficient densities of ions.

The Dual Laser Plasma (DLP) technique for photoabsorption spectroscopy [14, 15], addresses two of the principal challenges that arise in photoabsorption experiments, namely the generation of a sufficient column density of the species of interest, and creating a bright source of continuum radiation to probe these ions. This laboratory-scale experiment relies on lasers to produce plasmas, which are highly dynamic. The laser pulse first heats and boils the solid target, then ionises the material ablated to form a plasma. Two plasmas are generated, the first of which is populated with the ions of interest. The second plasma emits the continuum radiation used to probe these ions after a controlled time delay which allows the required ion population to evolve. Because the plasma expands and cools in the few $\mu$s after formation, increasing the time delay allows for lower ion stages to be accessed, while shorter delays make it possible for higher ion stages to be selected [16].

This ion population selectivity, based on initial laser parameters and the chosen inter-plasma time delay, makes the DLP method well suited to the study of neutral atoms and few times ionised species [17, 18]. Careful tuning of experimental parameters also facilitates studies of the structure of metastable states [19]. However, the majority of DLP photoabsorption studies have been limited at wavelengths longer than about 100 nm, as the quality of the continuum emitted by laser-produced plasmas diminishes above that wavelength [20].

Bright sources of continuum radiation in the visible and near- to mid-infrared regions of the spectrum have developed rapidly in the last two decades. The most promising for laboratory-scale experiments are supercontinuum lasers based on non-linear processes in optical fibres [21], and micro-structured optical fibres [22], which are pumped by either compact pulsed solid state lasers or fibre lasers. These lasers can deliver broadband continuum pulses (typically 1000’s of nm), delivering nano or microjoules of energy in ns-duration pulses at rates from a few hertz up to 100s of MHz. The optical pulses are inherently noisy on a shot-to-shot basis, and much effort is underway to improve spectral intensity stability.

These supercontinuum lasers are now commercially available, opening up a new field of experimental studies of ions and atoms generated in laser-produced plasmas. We show a new experiment combining a supercontinuum laser as a probe, and a laser-produced plasma ion source, generating photoabsorption spectra in the visible and near-infrared regions of the spectrum. The purpose of the experiment is to measure spectra of singly to quadruply ionised atomic species, providing data on transition energies and oscillator strengths to meet a wide variety of astronomical and astrophysical research needs.

Spectra of yttrium plasmas are presented to demonstrate the capabilities of the experiment. Known lines in near infra-red spectra of neutral and singly-ionised yttrium are listed on the NIST database [3], and in work by Nilsson et al. [23], who reported emission spectra of yttrium from a hollow cathode discharge plasma. These were used to aid the analysis in the current work.

## Development and method

**Fig. 1:**
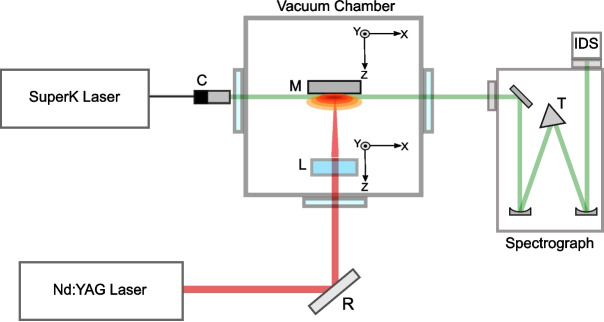
The schematic of the experimental setup for NIR absorption spectroscopy using the NKT SuperK super continuum laser as a probe. The vacuum chamber was fitted with three anti-reflection coated windows (IDS Camera, R: reflector, L: motorised cylindrical lens, C: laser collimator, M: motorised target mount, T: motorised triangular turret containing three different diffraction gratings)

A Q-switched Surelite III neodymium yttrium aluminium garnet (Nd:YAG) laser was used to generate the absorbing plasma, as displayed in Fig. 1. The laser was operated at 1064 nm with a repetition rate of 10 Hz and a pulse duration of 6 ns. The laser energy was measured to be 736 ± 6 mJ. A plano-convex cylindrical lens with a focal length of 10 mm was used to create a line plasma by focusing the Nd:YAG pulses at normal incidence onto a solid, planar yttrium target of 99${\%}$ purity. The target requires no special preparation, any initial oxide layer can be removed by a de-focused laser pulse. The line plasma formed was approximately elliptical in shape with a major axis (parallel to the optic axis of the spectrometer) measured to be 10 mm and a minor axis of 3 mm. The average power density across the focal spot was calculated to be 5.2 x 10$^{8}$ W cm$^{-2}$. Both the cylindrical lens and the target were connected to motorised stages, enabling motion in three linear axes. These allow the distance between the target and the lens and the distance of the target from the probe beam to be controlled with an accuracy of a few $\mu$m. Motorised stages were also used to ensure that a fresh area of target was presented, to prevent cratering following excessive laser irradiation.

An NKT Photonics SuperK COMPACT supercontinuum laser was the continuum source to probe the line plasma formed. This laser has a collimated output in the spectral range of 450 - 2400 nm, at a repetition rate that can be varied from 1 - 22 kHz. For these experiments the repetition rate was set at 10 Hz. The pulse length of the laser is 1.6 ns ± 0.1 ns, with energy per pulse measured to be 37 $\mu$J ± 0.1$\%$. The full width at half maximum (FWHM) beam waist diameter varies with wavelength and is of order 1.0 mm with a divergence of 1.0 mrad. The beam was aligned parallel to the target surface and passed through the line plasma at a given distance above the surface of the target metal, and hence at different column densities. In this experiment the collimated continuum beam probed the region between the flat target surface and a height of 500 $\mu$m above it.

The target and motors were located within an experimental chamber, which could be evacuated, but contained air at atmospheric pressure for the duration of these experiments. This yielded the highest signal to noise ratio in the spectra, due to confinement of the absorbing plasma. The chamber was fitted with three anti-reflection (AR) coated windows and an electrical feed-through which facilitated power and communication with the motors located inside the chamber. Spectra were recorded using an Acton SpectraPro 2750 Czerny-Turner spectrometer with a focal length of 750 mm fitted with a 300-groove/mm diffraction grating which was blazed for 1-$\mu$m radiation. The width of the entrance slit of the spectrometer was set to ${\sim }$0.05 mm. The spectra were imaged using an inexpensive complementary metal-oxide semiconductor (CMOS) camera (IDS model U3-3682XLE-NIR) with pixels measuring 2.2 $\mu$m x 2.2 $\mu$m, arranged in a 2592 x 1944 array. The linear dispersion at 800 nm is 4.33 nm/mm, providing a coverage of approximately 0.01 nm per pixel at that wavelength. The sensitive area of the camera measured 5.70 mm x 4.28 mm. The SuperK laser, the Surelite Nd:YAG laser, the target chamber and the Acton spectrometer were all secured onto an optical bench measuring 1.2 m x 2.4 m.

**Fig. 2:**
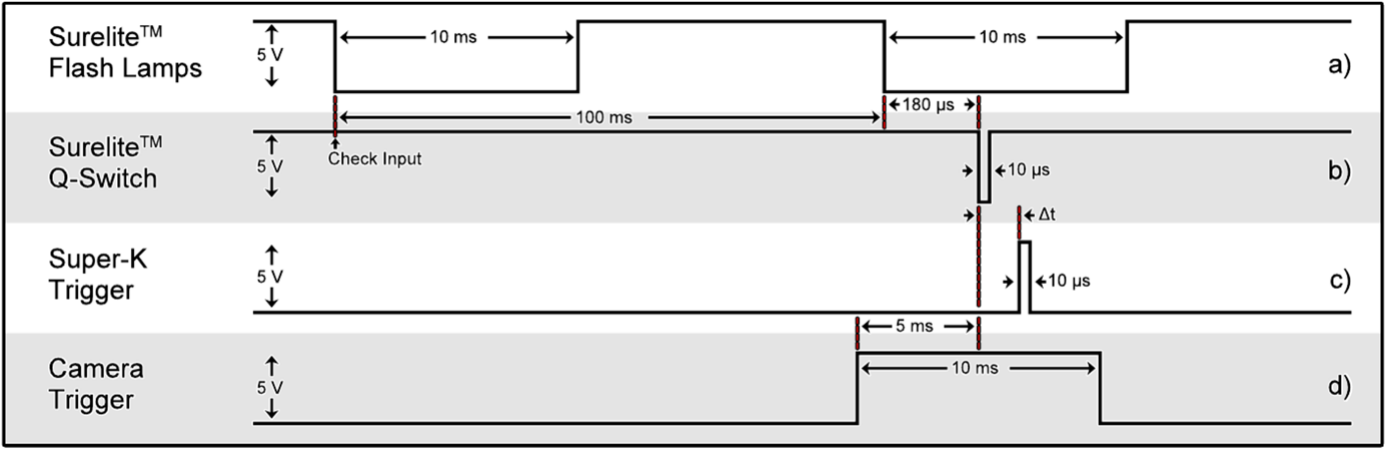
Electronics timing diagram for the camera and laser devices. Operation begins at "Check Input" upon receiving a command from the control application. The temporal axis is not to scale

The SuperK laser can be set to fire at a variable time delay ($\Delta$t) after the initial Nd:YAG pulse, see Fig. 2. This time delay was set to 11 $\upmu$s, with a jitter of ± 70 ns. As with all supercontinuum lasers, the output spectrum of the SuperK laser varies stochastically from shot to shot. Averaging over 750 shots was found to achieve a sufficient signal to noise ratio, in groups of 15 laser pulses per camera exposure repeated 50 times. For any group of 750 shots, the standard deviation of the noise was measured to be between 2 and 14 $\%$ of the amplitude of the average continuum signal, depending on spectral region. Fresh target material was presented to the laser after each 750 shots as there was very little ablation of the target due to the large spot size forming the absorbing plasma.

The $I_0$ signal (SuperK only), the *I* signal (SuperK through absorbing plasma) and $I_e$ signal (emission from the absorbing plasma) were recorded for each wavelength setting on the Acton spectrometer. The absorption spectrum was then calculated following the Beer-Lambert law, giving a spectrum of absorbance as a function of wavelength.

1 $$\begin{aligned} Absorbance = log_{10}\left( \frac{I_0}{I - I_e}\right) \end{aligned}$$

**Fig. 3:**
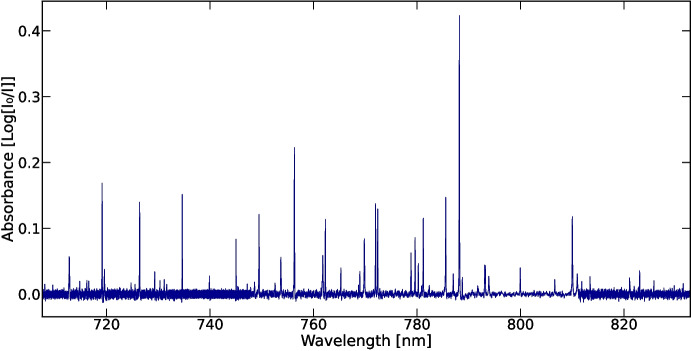
The photoabsorption spectrum of a yttrium laser-produced plasma recorded at a delay of 11 $\upmu$s in the range 708 - 832 nm. Due to the limited spectral range of the spectrometer, data from multiple overlapping measurements were combined to construct the full spectrum

The translation stages were motorised using Nema 8 captive linear actuators, each driven by a custom-designed integrated circuit incorporating a STSPIN820 microcontroller. Timing control of electronic signals triggering the Surelite and SuperK lasers was handled by an Arduino Nano A000005. These electronic systems were operated through a suite of purpose-built Python control software, featuring a graphical user interface to enable user-friendly operation.

The flashlamps of the Surelite laser were triggered at 10 Hz by a 10-ms wide 5-V signal. The Q-switch was activated by the control software to deliver a 10-$\upmu$s, 5-V signal 180 $\upmu$s after the rising edge of the flashlamp signal to achieve close to maximum population inversion. Subsequently, 11 $\upmu$s after the Q-switch, the SuperK laser output was triggered by a 10-$\upmu$s wide 5-V signal. The IDS camera captured images with a 10-ms exposure time, when triggered on the Q-Switch pulse.

Spectra were initially calibrated using a geometric calculation of the dispersion for a given grating angle setting to a position on the CMOS camera. The wavelength range was divided by the number of pixels present along the length of the camera to calculate the wavelength at each individual pixel. Fine calibration was performed using known spectral lines of yttrium listed in the Atomic Spectra Database of the National Institute of Standards and Technology (NIST) [3] and the work of Nilsson et al. [23]. A second-order polynomial function using least-squares regression was fitted to obtain a calibration curve for each individual spectrum. The residuals of identified lines were of the order of $10^{-3}$ to $10^{-4}$ nm, and the accuracy of the line identification was estimated to be of order ± 0.01 nm. Spectra were recorded in $\sim$20 nm bands, with typically 1.5 nm overlap at each end. Small variations in baseline levels were corrected by fitting a locally weighted estimated scatterplot smoothing (LOWESS) curve to each individual spectrum and subsequently subtracting it. Individual spectra were then combined to produce the final spectrum shown in Fig. 3.

## Results and discussion

A series of spectra were recorded between 700 nm and 1100 nm. Spectra between 708 nm and 832 nm are presented here. This region was chosen as many of the lines observed are reported in the NIST database [3] which aided both calibration and our assessment of the technique. There are very few lines beyond 850 nm in the NIST holdings for yttrium.

As can be seen in Fig. 3, the spectrum in this range contains many distinct lines with a maximum height of the absorbance of 0.42 on a noise floor of typically ±0.01 units. The FWHM of spectral lines at the best performance of the spectrometer was measured to be 0.071 nm. The FWHM of the experimentally found yttrium absorption lines varies from 0.072 - 0.111 nm, due to plasma and atomic broadening effects. The majority of the observed lines are from the spectrum of Y I, while five transitions from $4d^{2}$ metastable levels and one $4d6p(^{1}$D$_{2}$) - $4d6d (^{1}$F$_{3}$) transition, at 821.08 nm, in the Y II spectrum are also observed. The majority of the resonance lines in the spectrum of Y II are known to lie at shorter wavelength [3]. No lines arising from transitions in the spectrum of Y III [24] were observed. The observed ion population consists of mainly neutral yttrium with a smaller population of singly-ionised yttrium. This population distribution is consistent with previous studies of laser-produced plasmas using extreme ultraviolet (EUV) photoabsorption spectroscopy [14, 25]. In these works, plasmas are seen to be dominated by neutral species at time delays of order 1000 ns, with some evidence of singly ionised species in the spectra.

The plasma electron temperature as a function of power density can be estimated using the empirical formula given by Colombant and Tonon [26], and later modified by Wong et al. [27] as:

2 $$\begin{aligned} T(eV) = 5.2 \times 10^{-6}A^{0.2}(\lambda ^{2}\phi )^{0.6} \end{aligned}$$

where A is the atomic number of the atom dominating the plasma, $\lambda$ is the laser wavelength in $\mu$m, and $\phi$ is the laser power density in $W/cm^{2}$. This gives a value of 1.8 eV for the plasma electron temperature at the time of formation. Expansion and cooling of the plasma over the 11 $\upmu$s before being probed by the supercontinuum laser pulse saw this temperature drop to below 0.5 eV, as evidenced by previous experiments [25, 28]. This experimental setup has also been used to measure absorption in other elements, including zirconium, platinum and gold, but whose analysis is not presented here. Notably, the spectrum of gold and platinum were line poor within the same spectral range.

As this experiment was conducted at atmospheric pressure, the expansion of the plasma plume was impeded compared to that of a plasma created under vacuum conditions. The interaction with the ambient atmosphere in the chamber [29] has been shown to decelerate the motion of the plasma plume. This maintains the plasma closer to the target surface which enhanced the observed absorption at time delays of 1000 ns and longer. Futhermore, previous studies of shockwave dynamics of laser-produced plasmas in atmospheres at greater than 100-mbar pressure have found that at times out to 1000 ns, oxygen molecules are displaced away from the target surface [30]. Thus, the presence of molecular absorption features among the unidentified lines within the observed spectra has been ruled out.

Table 1 provides 26 previously identified Y I and Y II lines, from either the NIST database, or work by Nilsson et al. [23]. There is reasonable agreement between the absorbances measured in this experiment and the reported intensities from previous works. In addition, 29 lines not previously identified are listed in Table 2, with their absorbance peak height also shown. Four of these lines are weak, with peak absorbance values of 0.01.

**Table 1:** List of previously identified yttrium absorption lines observed in this work between 708 nm and 832 nm, with assignments

$\varvec{\lambda _{\mathrm {Exp.}}}$ [nm]	Absorbance	Previous Work [nm]	Reported Intensity	Ion	Transition		
712.80	0.06	712.7970$$^{a}$$	11$$^{a}$$	I	4d5s($$^{1}$$D)5p($$^{2}$$D$$_{3/2}$$)	-	4d$$^3 $$ ($$^{2}$$F$$_{5/2}$$)
719.16	0.17	719.16404$^{a}$	35$^{a}$	I	4d5s($^{3}$D)5p($^{4}$F$_{7/2}$)	-	4d$^3$ ($^{4}$F$_{7/2}$)
719.60	0.04	719.5874$^{a}$	10$^{a}$	I	4d5s($^{1}$D)5p($^{2}$F$_{5/2}$)	-	4d$^3$ ($^{2}$F$_{7/2}$)
726.41	0.14	726.4159$$^{a}$$	35$$^{a}$$	II	4d$$^2$$($$^{1}$$D$$_2$$)	-	4d5p($$ ^{3}$$D$$_1$$)
729.31	0.03	729.3073$^{a}$	9$^{a}$	I	4d5s($^{3}$D)5p($^{4}$F$_{7/2}$)	-	4d$^3$ ($^{4}$F$_{5/2}$)
734.63	0.19	734.6457$^{a}$	50$^{a}$	I	4d5s($^{3}$D)5p($^{4}$F$_{9/2}$)	-	4d$^3$ ($^{4}$F$_{9/2}$)
739.88	0.03	739.8712$^{a}$	11$^{a}$	I	4d5s($^{1}$D)5p($^{2}$F$_{7/2}$)	-	4d$^3$ ($^{2}$F$_{7/2}$)
745.03	0.08	745.0275$^{a}$	29$^{a}$	II	4d$^2$($^{3}$P$_2$)	-	4d5p($^{1}$P$_1$)
749.48	0.12	749.4848$^{a}$	17$^{a}$	I	4d5s($^{3}$D)5p($^{4}$P$_{3/2}$)	-	4d$^3$($^{4}$P$_{5/2}$)
756.31	0.22	756.31149$^{a}$	35$^{a}$	I	4d5s($^{3}$D)5p($^{4}$P$_{5/2}$)	-	4d$^3$ ($^{4}$P$_{5/2}$)
761.79	0.06	761.7608$^{a}$	8$^{a}$	I	4d$^{2}$($^{3}$F)5p($^{4}$G$_{9/2}$)	-	4d$^{2}$($^{3}$F)6s($^{4}$F$_{7/2}$)
762.29	0.11	762.2961$^{a}$	19$^{a}$	I	4d5s($^{3}$D)5p($^{4}$P$_{1/2}$)	-	4d$^3$ ($^{4}$P$_{3/2}$)
765.29	0.04	765.2854$^{a}$	7$^{a}$	I	4d5s($^{3}$D)5p($^{4}$P$_{3/2}$)	-	4d$^3$ ($^{4}$P$_{3/2}$)
768.95	0.03	768.9472$^{a}$	5$^{a}$	I	4d5s($^{3}$D)5p($^{4}$P$_{1/2}$)	-	4d$^3$ ($^{4}$P$_{{1/2}}$)
771.99	0.14	771.9888$^{a}$	19$^{a}$	I	4d5s($^{3}$D)5p($^{4}$P$_{3/2}$)	-	4d$^{3}$ ($^{4}$P$_{1/2}$)
772.40	0.13	772.4057$^{a}$	19$^{a}$	I	4d5s($^{3}$D)5p($^{4}$P$_{5/2}$)	-	4d$^{3}$ ($^{4}$P$_{3/2}$)
778.84	0.06	778.8402$^{a}$	13$^{a}$	I	4d5s($^{3}$D)5p($^{4}$D$_{1/2}$)	-	4d$^{3}$ ($^{4}$F$_{3/2}$)
779.63	0.09	779.6291$^{a}$	13$^{a}$	I	4d5s($^{3}$D)5p($^{4}$D$_{3/2}$)	-	4d$^{3}$ ($^{4}$F$_{5/2}$)
780.25	0.05	780.2487$^{a}$	6$^{a}$	I	4d$^2$($^{3}$F)5s($^{2}$F$_{5/2}$)	-	4d5s($^{1}$D)5p($^{2}$P$_{3/2}$)
781.21	0.16	781.2132$^{a}$	17$^{a}$	I	4d5s($^{3}$D)5s($^{4}$D$_{5/2}$)	-	4d$^3$ ($^{4}$F$_{7/2}$)
785.55	0.15	785.5468$^{a}$	29$^{a}$	I	4d5s($^{3}$D)5p($^{4}$D$_{7/2}$)	-	4d$^3$ ($^{4}$F$_{9/2}$)
788.19	0.42	788.1878$^{a}$	110$^{a}$	II	4d$^2$($^{1}$D$_2$)	-	4d5p($^{1}$P$_1$)
799.94	0.04	799.9358$^{a}$	10$^{a}$	I	4d5s($^{3}$D)5p($^{4}$D$_{7/2}$)	-	4d$^3$ ($^{4}$F$_{7/2}$)
806.61	0.02	806.6092$^{b}$	10$^{b}$	II	4d$^2$($^{1}$D$_2$)	-	4d5p($^{3}$F$_{2}$)
821.08	0.03	821.0852$^{b}$	27$^{b}$	II	4d6p($^{1}$D$_{2}$)	-	4d6d($^{1}$F$_{3}$)
829.70	0.01	829.7042$^{b}$	4$^{b}$	II	4d$^{2}$($^{3}$P$_{2}$)	-	4d5p($^{1}$D$_{2}$)

$^{a}$From the NIST Atomic Spectra Database $^{b}$From Nilsson et al. (1991). Note: the intensities, in arbitrary units, reported by Nilsson are typically 3x those for the same lines in the NIST database

**Table 2:** List of unidentified yttrium absorption lines observed in this work between 708 nm and 832 nm

Wavelength [nm]	Absorbance
708.07	0.02
709.67	0.01
712.85	0.06
714.82	0.02
716.21	0.02
716.58	0.02
724.76	0.02
725.52	0.02
730.33	0.02
731.15	0.02
731.64	0.02
747.85	0.02
748.60	0.02
752.56	0.02
753.69	0.06
768.69	0.01
780.87	0.01
782.38	0.01
787.00	0.03
788.76	0.03
791.71	0.02
793.08	0.04
793.20	0.04
793.87	0.03
810.01	0.12
810.92	0.03
813.43	0.03
823.02	0.04
825.77	0.02

This experiment is a demonstration of the technique of near-infra-red photoabsorption using laser-produced plasmas to generate populations of neutral species and ions. The novel step is the deployment of a supercontinuum laser as the backlighting continuum. Previously the DLP technique has been mostly limited to the soft x-ray and EUV regions of the spectrum, below 50 nm, by the relative ease of generating a laser-produced plasma continuum at these wavelengths. Using supercontinuum lasers, as shown here, opens up the possibility of performing laboratory astrophysical experiments to record photoabsorption spectra of ions, with great selectivity over the temporal and spatial characteristics of the plasma. The spectral range accessible to the technique is between 450 nm and 4000 nm, and possibly beyond these limits, depending on the equipment chosen. In future experiments, the absorbing laser-produced plasma can be characterised using EUV and soft x-ray DLP studies to establish the temporal and spatial distributions of the species under study, and time-resolved emission spectroscopy to estimate plasma temperatures and electron densities. Improvements to the setup are planned in a number of areas, including fitting a short-wavelength infrared (SWIR) camera with spectral coverage to 1700 nm, and a laser timing system which will reduce the effective jitter from ±70 ns to ±2 ns. This will facilitate the use of shorter time delays to record spectra of a larger range of ion stages.

## Conclusion

We have shown a novel experiment which will provide broad-wavelength photoabsorption spectra of near-neutral species, initially with an interest in the interpretation of kilonova spectra, but with applications to other fields of astrophysics, and potentially in other disciplines. We report clear absorption lines in neutral and singly ionised yttrium, with 26 known lines and 29 unknown lines observed between 708 nm and 832 nm. The stochastic nature of the continuum spectrum emitted by the supercontinuum laser has been managed in this experiment by summing 750 shots to form a spectrum. With this technique, any solid element that can be formed into a stable, planar or cylindrical target, can be explored. We can achieve a plasma dominated by a single ion stage by controlling the laser focus, the region of plasma probed, and the time delay between plasma formation and probe pulse. This wide parameter space allows up to quadruply ionised atomic species to be accessed. Future developments with this laboratory-scale experiment will involve reduced time delay jitter, fewer shots required per spectrum, and wavelength coverage out to 1700 nm.

## Data Availability

Data is provided within the manuscript. The raw absorbance data for Fig. 3 is available on Zenodo. 10.5281/zenodo.15231188 Hyperlink: https://zenodo.org/records/15231188
